# Electromagnetic hypersensitivity: a critical review of explanatory hypotheses

**DOI:** 10.1186/s12940-020-00602-0

**Published:** 2020-05-06

**Authors:** Maël Dieudonné

**Affiliations:** 1 Max Weber Center, Institut des Sciences de l’Homme, 14 avenue Berthelot, F-69007 Lyon, France; 2 Health Services and Performance Research, University Hospital of Lyon, 162 avenue Lacassagne, F-69003 Lyon, France

**Keywords:** EMF health effects, Functional somatic syndromes, Health beliefs, IEI-EMF, Media effects, Modern health worries, Nocebo response, Risk perception

## Abstract

**Background:**

Electromagnetic hypersensitivity (EHS) is a condition defined by the attribution of non-specific symptoms to electromagnetic fields (EMF) of anthropogenic origin. Despite its repercussions on the lives of its sufferers, and its potential to become a significant public health issue, it remains of a contested nature. Different hypotheses have been proposed to explain the origin of symptoms experienced by self-declared EHS persons, which this article aims to review.

**Methods:**

As EHS is a multi-dimensional problem, and its explanatory hypotheses have far-reaching implications, a broad view was adopted, not restricted to EHS literature but encompassing all relevant bodies of research on related topics. This could only be achieved through a narrative approach. Two strategies were used to identify pertinent references. Concerning EHS, a complete bibliography was extracted from a 2018 report from the French Agency for Food, Environmental and Occupational Health & Safety and updated with more recent studies. Concerning related topics, the appropriate databases were searched. Systematic reviews and expert reports were favored when available.

**Findings:**

Three main explanatory hypotheses appear in the literature: (1) the electromagnetic hypothesis, attributing EHS to EMF exposure; (2) the cognitive hypothesis, assuming that EHS results from false beliefs in EMF harmfulness, promoting nocebo responses to perceived EMF exposure; (3) the attributive hypothesis, conceiving EHS as a coping strategy for pre-existing conditions. These hypotheses are successively assessed, considering both their strengths and limitations, by comparing their theoretical, experimental, and ecological value.

**Conclusion:**

No hypothesis proves totally satisfying. Avenues of research are suggested to help decide between them and reach a better understanding of EHS.

## Introduction

Electromagnetic hypersensitivity (EHS) is a condition defined by the attribution of non-specific symptoms to electromagnetic fields (EMF) of anthropogenic origin. Its sufferers predominantly report sleep disorders, asthenia, headaches, memory and concentration difficulties, dizziness, musculoskeletal pain, skin conditions and mood disorders, for which they hold responsible EMF emitted by various devices, including mobile phone base stations and mobile handsets, Wi-Fi routers, DECT telephones, household appliances, compact fluorescent and halogen light bulbs, power lines and power transformers, or smart meters [1–6]. EHS is also characterized by specific and sometimes spectacular behaviours, that intermittently draw attention from the media and make it known from the public. These include getting rid of personal electronic devices, avoiding highly exposed places like shopping centres or public transports, wearing EMF-shielding clothes, sleeping under protective canopies, or taking shelter in isolated woods or caves [7–9].

The appearance of EHS is undocumented. The first obvious cases of EHS were reported in Sweden in the late 1980s, and were first referred to as hypersensitivity to electricity [10, 11], then as electromagnetic hypersensitivity [12]. According to some sources, the history of EHS goes back further to the 1980s, with the reporting of dermal reactions by video display terminal workers [13], or even to the 1950s, with the observation of various health issues among radio and radar technicians, especially in the Soviet Union [14]. However, data corroborating this second hypothesis is sparse [15, 16]. In France, the first cases were identified in 2006, and their number has been growing steadily since [17]. As an indication, as of April 10th, 2020, 1667 people had identified themselves as EHS to the nationwide French organization Une Terre pour les EHS. It is possible that the number of EHS persons is actually much higher, given both the socio-cognitive challenges involved in recognizing one’s hypersensitivity and the multiplication of radio-transmitting devices in the everyday environment (see below). Indeed, available epidemiological data points to a relatively high prevalence of perceived EMF sensitivity in the general population, reaching 1,6% in Finland and 2,7% in Sweden, 3,5% in Austria, 4,6% in Taiwan, 5% in Switzerland and 10.3% in Germany [18–22].

Despite its repercussion on the lives of the affected persons [23] and its potential to become a significant public health issue, EHS still eludes the efforts to objectify it. It remains a self-diagnosed condition, without an objective case-definition [24], while the origins of symptoms experienced by its sufferers are still contested and unclear. That situation led experts for the World Health Organization, over 10 years ago, to recommend abandoning the term EHS in favor of an etiologically neutral designation: Idiopathic Environmental Intolerance attributed to EMF [25]. Different explanatory hypotheses have been proposed in the literature, which this article aims to analyze critically. It is not a systematic review of the literature on EHS, because the assessment of its purported causes requires a broad view, encompassing distinct bodies of research from various disciplines, which can only be achieved through a narrative approach. These bodies of research relate e.g. to EMF biological and health effects, the determinants of symptoms perception, modern health worries, protests against the siting of mobile phone base stations, or functional somatic syndromes. Two strategies were used to identify relevant references. Concerning EHS, a complete bibliography was extracted from a 2018 report from the French Agency for Food, Environmental and Occupational Health & Safety [26] and updated with more recent studies. Concerning related topics, the appropriate databases were searched. Systematic reviews and expert reports were favored when available.

Upon examination of the literature, three main explanatory hypotheses of EHS symptoms came to light: (1) the electromagnetic hypothesis, attributing EHS to EMF exposure; (2) the cognitive hypothesis, assuming that EHS results from false beliefs in EMF harmfulness, promoting nocebo responses to perceived exposure; (3) the attributive hypothesis, conceiving EHS as a coping strategy for pre-existing conditions. These hypotheses are assessed successively, considering both their strengths and limitations, by comparing their theoretical, experimental, and ecological value. The implications for future research are discussed in conclusion.

## The electromagnetic hypothesis

### First argument: EHS persons’ attributions

The most straightforward cause of EHS symptoms is EMF exposure, as reported by EHS persons. Several studies documented their attributions, which have not been systematically reviewed. The most regularly blamed devices seem to be mobile telephony base stations and handsets, cordless phones, personal computers, TV sets, microwaves oven, and power lines. However, the frequency of complaints related to each of these devices varies significantly between studies, e.g. from 12,9 to 80% of the sample for base stations, 11 to 71% for cordless phones, 28,4 to 79% for power lines, etc. [1–4, 6]. These discrepancies might reflect a natural variability among EHS persons, or differences in the questionnaires used as well as in the respondents’ electromagnetic environment.

### Second argument: recent increase of RF EMF exposure

The attributions of EHS persons are consistent with the recent increase of RF EMF exposure in the general population, resulting from the multiplication of wireless communication technologies and devices. In France, for instance, mobile telephony was introduced in 1986 before gaining momentum in the late 1990s: more than 200,000 base stations are now deployed on the metropolitan territory, while almost 95% of people aged 12 and over own a handset [27]. Similarly, digital cordless phones were introduced from 1992 onward and Wi-Fi devices from 1999 onward, following the publication of the relevant standards of communication, and are now widespread in domestic and professional environments. It is therefore undisputable that EMF exposure has increased massively in the high frequency bands of the electromagnetic spectrum (3 MHz – 30 GHz) used by these technologies. This justifies paying close attention to RF EMF purported health risks: even the weakest biological effects could have disastrous sanitary consequences, given that virtually all of the general population of developed countries is continuously exposed nowadays. Regarding LF EMF exposure, there is no indication of a similar phenomenon. Technologies and devices generating EMF in the low frequency bands of the electromagnetic spectrum (3 Hz – 300 kHz) are mostly associated with the production, transportation, and consumption of electricity. They became widespread long before the onset of EHS.

### First limitation: a low biophysical plausibility

The electromagnetic hypothesis suffers from a low biophysical plausibility: proven ELF and RF EMF biological effects cannot explain the symptoms experienced by EHS persons because (1) their health consequences are qualitatively different, and (2) the levels of exposure required for such consequences to appear are much higher than those actually observed in daily environments.

RF EMF have a demonstrated capacity to heat up organic matter through the alternative polarization of water molecules. When exceeding the thermoregulating potential of living organisms, such thermal effects might induce superficial or deep burns, notably of the weakly vascularized crystalline lens, leading to cataracts, or a general hyperthermia [28]. These are not typical EHS symptoms. Furthermore, the levels of RF EMF exposure of the general population are vastly insufficient to provoke thermal effects: in Europe, depending on countries and exposure assessment methods, they range between 0,16 and 0,29 V/m at home and between 0,2 and 0,76 V/m in outdoor environments [29, 30], while levels deemed safe for continuous exposure range between 28 and 87 V/m [31]. Research into “non-thermal” RF-EMF effects remains inconclusive to date, notably because studies with positive findings often suffer from serious methodological limitations, making false positives likely [32]. A noteworthy exception concerns sleep electroencephalography: half a dozen studies has observed that RF EMF exposure triggers an increased spectral power of so-called sleep spindles [33–35]. However, that increase is not associated with any alteration of sleep macrostructure or perceived sleep quality, was not found to be higher among EHS persons than healthy controls [36], while several ecological studies did not find any correlation between RF EMF exposure and sleep disorders [37–39]. Thus, although this phenomenon is intriguing and warrants further investigation, it remains an unlikely explanation for EHS symptoms at this time.

LF EMF have a demonstrated capacity to create electrical currents within the body through a phenomenon of electromagnetic induction. These might stimulate excitable tissues, involved in the propagation of nervous signals, provoking phosphenes or paresthesia. At higher intensities, induced currents can have similar effect to an electrocution, including deep burns and ventricular fibrillation [28]. Such symptoms are different from those of EHS. Moreover, for them to appear, significantly higher levels of LF EMF exposure are necessary than those actually observed: in Europe, they range between 0,01 and 0,1μT in average, while only 0,5% of the general population is exposed to levels above 0,2 μT for significant periods, because of residential proximity to intense sources like power lines and mass transports [40]. For reference, a level of 200 μT is deemed safe at a frequency of 50 Hz, accounting for the majority of LF EMF exposure in Europe [41]. Significantly higher levels of exposure can be reached in occupational settings, but there is no indication that EHS persons hold the relevant jobs (involving induction furnaces, arc welding, medical imaging, etc.). Other LF EMF biological and health effects were investigated without conclusive results. The notable exception is childhood leukemia, which several epidemiological studies found associated with residential proximity to power lines, at very low levels of exposure (> 0,4 μT) [42]. However, this association is not corroborated by in vitro results [43] and is not apparent in studies using direct measures of LF EMF exposure, rather than the distance to power lines as a proxy [44–46]. Therefore, this finding is increasingly regarded as resulting from selection bias (e.g., participation rates are often higher for cases than control) or hidden correlations with living conditions (e.g., exposure to benzene from road traffic, as power lines tend to be located near highways).

The observation of such low levels of exposure in the general population might seem surprising, given the high number of RF and LF EMF emitting devices in today environments. One explanation is that exposure induced by such devices decreases squarely by distance (i.e., they must operate in close proximity to the body to generate biologically significant exposure). Another is that their commercialization and exploitation is tightly regulated in developed countries, with the aims of preserving electromagnetic compatibility and preventing the occurrence of proven biological effects [31, 41]. It remains possible to argue that yet undiscovered EMF health effects might appear below regulatory levels of exposure and explain EHS symptoms. Indeed, it is scientifically impossible to disprove the existence of a risk in absolute terms: this residual uncertainty arguably contributes to many controversies in environmental health, as it can always justify a precautionary approach [47]. On the other hand, given the immense wealth of EMF research, and the incredibly widespread use of RF and LF EMF emitting technologies, it can be argued that any other biological effect than dielectric heating and electromagnetic induction would have been already discovered by serendipity [15, 16].

Lastly, a few pathogenic mechanisms were hypothesized to explain how EMF exposure could cause EHS symptoms, none of which was rigorously proven. In particular, EMF was proposed to affect living organisms by activating voltage-gated calcium channels, which could in turn enhance the production of nitric oxide and provoke various health effects [48]. This assumption is vindicated by the observation of increased levels of blood biomarkers of oxidative stress among EHS persons [49]. However, a supplementary antioxidant therapy did not reduce EHS persons’ symptoms [50], while in vivo and in vitro studies did not find conclusive evidence of an oxidative effect of EMF exposure [51]. It thus remains impossible to explain, from a biophysical perspective, how EMF exposure could cause EHS symptoms.

### Second limitation: the failure of experimental studies

Most studies of EHS are experimental, consisting in intentionally exposing EHS persons to EMF in order to observe their reactions. These so-called provocation studies offer the most rigorous way of assessing EHS purported electromagnetic etiology, as they allow, when conducted in shielded rooms, to precisely control EMF exposure and thus to isolate its effects. However, in double blind conditions, they failed to establish a link between such exposure and EHS persons’ subjective assessments (feeling of being exposed, number and intensity of reported symptoms, etc.) [52] and physiological responses (heart-rate variability, skin temperature and conductance, blood chemistry, sleep electro-encephalography, etc.) [53].

Consequently, several systematic reviews concluded that EHS persons likely do not react to EMF exposure, notwithstanding provocation studies’ methodological limitations [54]. In particular, their statistical power is usually insufficient, leading to imprecise results. They cannot ensure the homogeneity of test and control groups, in the absence of an objective case-definition of EHS, which may conceal the reactions of genuine EHS persons. Furthermore, they focused on a limited range of exposure types, mostly related to mobile telephony and 50/60 Hz electricity, neglecting many devices to which EHS persons attribute their symptoms. They also seldom involved mixed signals, comparable to EMF exposures in daily life, and focused on short-term and acute effects, to the detriment of possible cumulative and chronic effects. Therefore, the negative conclusion drawn from provocation studies might appear somewhat premature.

Several recent studies tried to address these limitations with innovative experimental protocols. They used portable exposure devices to conduct the tests at the subjects’ home, so as to reduce their level of stress and to eliminate potential exposure while traveling to a laboratory, while preserving double-blind conditions. These studies also included subjects asserting to react to EMF within the timeframe of the experiment, and performed statistical analysis at the individual level, but none of their 45 subjects reacted differently to real and sham exposure [55, 56]. Thus, experimental results remain inconsistent with the electromagnetic hypothesis.

### Third limitation: the uncertain results of environmental studies

Other studies took an ecological approach, assessing the relationship between EMF exposure and EHS symptoms in daily life. Such studies face significant metrological challenge, as there is no straightforward way of characterizing EMF exposure. They must resort to various methods, ranked by increasing quality as follows: subjective assessment, geocoded distance from mobile base stations, power lines, etc., geospatial propagation modelling, spot measurement in relevant places, and continuous measurement with personal dosimeters [57]. Even that last method is imperfect, as personal dosimeters cannot measure near-field exposure, which must be assessed from self-reported use or data from wireless carriers, may not offer enough temporal resolution to distinguish between mean and peak exposure, are susceptible to body shielding, etc. Therefore, potential confounding factors must be carefully considered when interpreting results from environmental studies (e.g., EMF exposure may vary significantly with location and activities, which may independently influence the feeling and reporting of symptoms).

In the general population, a meta-analysis of 22 such studies found no significant association between EMF exposure and non-specific physical symptoms, after the exclusion of 21 additional studies of insufficient quality (especially for lack of adjustment for confounding variables) [58]. In the EHS population, only four environmental studies were conducted. Using a cohort design, the first observed no relationship between modelled EMF exposure at baseline, deemed an acceptable proxy of long-term residential exposure, and health outcomes after one year [59]. The second study found no association between self-reported and medically registered symptoms, on the one hand, and modelled EMF exposure on the other hand, except for isolated correlations with the use of electric blankets, induction hobs and electric chargers near headboards [60]. The last two studies resorted to a temporal and individual approach: their subjects carried personal dosimeters and filed symptoms diaries, allowing to assess whether changes in EMF exposure were followed by changes in symptoms in a definite timeframe. The first of these studies observed weak and disparate correlations in 4 out of 7 subjects, concerning mostly Wi-Fi exposure, which was followed by decreasing headache intensity in 2 subjects and increasing feeling of malaise in 1 subject [61]. The second study found a significant association in only 1 out of 36 subjects, after adjusting for confounders, between Wi-Fi exposure and the intensity of the most important complaint [62].

These mostly negative results could ensue from methodological inadequacies, notably related to exposure characterization (rates of change could be more relevant than mean or peak levels) and time frames of analysis (which could diverge from the duration necessary for EHS symptoms to occur). Still, they indicate that if a relationship between EHS symptoms and EMF exposure awaits discovery, it shall not be straightforward.

## The cognitive hypothesis

Depending on the relative importance given to these results and their limitations, the electromagnetic hypothesis may appear either still unproven or largely refuted. It remains possible that only a few EHS persons are actually sensitive to EMF, whose reactions are unobservable at a collective level, especially given the few positive results of environmental studies using an individual approach. However, these results are likely to reflect residual confounding or false positives, as (1) they are heterogeneous and partially contradictory, (2) are opposed to experimental studies conducted even at the individual level, and (3) are biophysically unexplained if not implausible. It might also be argued that the electromagnetic etiology of EHS is so complex it has not been adequately studied to date. But this assumption contradicts the attributions of EHS persons, some of which claim to react instantly to sources of exposure they have identified precisely: if such straightforward causal relations were true, it seems unlikely they would never have been observed unequivocally in either experimental or environmental studies. As a consequence, the attributions of many EHS persons are probably wrong. An alternative hypothesis was thus put forward, presenting them as causing EHS symptoms through nocebo responses to perceived EMF exposure.

### First argument: results from experimental and environmental studies

This cognitive hypothesis is supported by results from experimental studies, where EHS persons’ reactions often appear correlated with perceived exposure [52, 54] and where healthy subjects’ reactions to sham exposure can be amplified by the provision of alarmist information on EMF health effects [63–65]. These results demonstrate that the determinants of EHS persons’ reactions in laboratory settings are cognitive rather than biophysical in nature. Likewise, in environmental studies of EHS symptoms and of non-specific physical symptoms in the general population, the subjects’ complaints appear mostly independent from estimated or measured exposure, but significantly correlated with perceived exposure [58, 60, 62, 66]. As it appears, the more EHS persons believe to be exposed, the sicker they are, irrespectively of their actual exposure.

### Second argument: a comprehensive model

EHS symptoms may thus result from erroneous beliefs in the harmfulness of EMF. To explain how, a comprehensive model of EHS was recently proposed, drawing from the Bayesian predictive coding approach of brain functioning [67]. This models rests on the assumption that the brain is not a passive receiver of information, i.e., of sensory inputs from the body and the environment. Rather, it uses a prior model of the world to generate inferences on expected sensory inputs, which it constantly compares to actual inputs. When discrepancies arise, the brain can produce a new (posterior) model of the world, change the way it attends to sensory inputs, or alter behaviors (e.g., to generate more inputs compatible with the prior model or grant more weight to such inputs).

In the case of EHS, the prior model is characterized by a strong belief in the harmfulness of EMF, which leads the brain to pay extra attention to environmental cues related to them. When such cues are present while imprecise bodily signals are received, resulting e.g. from hypothalamic pituitary adrenal activation or stress-related hyperventilation, the brain is more likely to interpret them as symptoms provoked by EMF exposure. Conversely, when such signals are received while environmental cues are absent, the brain is more likely to look for them through confirmatory behaviors. This self-reinforcing process leads to a situation where the posterior model is totally shaped by the prior model, and symptoms can be experienced even without any physiological disturbance, as in nocebo responses.

EHS is thus explained analogically to hypochondria, classically attributed to a phenomenon of somato-sensory amplification [68], by a model where misplaced fears are no longer related to the body but to the environment. The strengths of this model are that it relies on accurate descriptions of the underlying brain processes, and is arguably applicable to other environmental intolerances: to chemicals [67], wind turbines [69], and possibly also power lines [70].

### First limitation: a dubious interpretation of experimental results

However, such an analysis rests on a disputable interpretation of experimental results. The observation that subjects believing in the harmfulness of EMF, whether because they regard themselves as EHS or have just received alarmist information, react adversely to perceived EMF exposure, actually demonstrates two things: that nocebo responses can be induced experimentally, and that EHS persons are susceptible to them. This is to be expected, as it merely exemplifies the phenomenon of suggestion, which is not specific to EHS. Proving that nocebo responses do occur among EHS persons in real-world contexts and are effectively responsible for their symptoms would require at least a follow-up of healthy subjects receiving alarmist information, to assess how lasting are the induced beliefs in EMF harmfulness, and their amplifying effect on reactions to sham exposure. This has never been done, i.e., there is no experimental evidence that the reception of alarmist information about EMF can durably alter prior models of the world and lead even to mild forms of EHS. Additionally, no indication appears in the literature that EHS persons are particularly susceptible to nocebo responses. Studies of their psychological characteristics mostly reported higher scores of depression or anxiety in comparison to healthy subjects [5, 71–77], which are not considered as enhancing nocebo responsiveness [78]**.**

### Second limitation: origins and effects of EMF worries

Furthermore, while the cognitive hypothesis grants a triggering role to erroneous beliefs, it does not convincingly explain their acquisition, i.e., how certain individuals become intimately convinced that EMF are harmful. Media reports are usually blamed, as they tend to seriously misrepresent health risks [79, 80], including about EMF [81–83] and EHS [84, 85]. This assumption credits them with a considerable influence on personal beliefs, implicitly going back to he “hypodermic needle” model of mass communication proposed by Laswell in 1927, in which they can literally inoculate opinions to the public. However, decades of research on mass communication have established this is certainly not the case, as the media repeatedly proved virtually powerless to change personal beliefs [86].

One reason pertains to the selectivity of media exposure: people strongly favor the media spreading information they already agree with, so as to avoid cognitive dissonance and reduce the cognitive load of processing data conflicting with their views [87]. This phenomenon might explain why the belief in EMF harmfulness tend to cluster with other “modern health worries”, which are themselves associated with health consciousness, positive views of alternative medicines, and even the interest for spirituality [88, 89]. These variables might reflect various traits of a specific subculture of Western societies, namely, the New Age movement, which is also characterized by a focalisation on the inner self as the source of truth, an holistic and esoteric way of thinking, a general defiance towards institutions and especially medicine, etc. [90, 91]. Another reason why the media fails to change personal beliefs pertains to reception. As their brain, individuals are not passive recipients of information: they possess critical skills allowing them to distance themselves from media reports, and to react with such attitudes as denial, skepticism, or indifference. Thus, as is now being acknowledged by risk perception studies, there are significant gaps between receiving information on the harmfulness of a technology, accepting it in principle, and feeling directly and personally threatened [92].

An illustration of this fact is found in studies of protests against the siting of mobile phone base stations. These consistently demonstrate that people act out of environmental and political concerns, related e.g. to the detrimental visual effect of base stations or the feeling of exclusion from the decision process leading to their siting. Protesters usually discover their alleged harmfulness in a second phase, when looking for arguments to consolidate their position against local authorities. Following the cognitive hypothesis, these activists are prime candidates to become EHS, as they express worries about EMF safety and have salient sources of exposure in their surroundings. However, this does not seem to be the case. Studies of protests against the siting of mobile phone base stations almost never mention EHS, unless about individuals already regarding themselves as such when these protests began. They also never allude to a degradation of the protesters’ health, nor to an alteration of their behaviors to systematically reduce their EMF exposure. The protesters’ concerns about EMF safety remain abstract, only reaching a personal level when linked with pre-existing conditions, like cancer or headaches, of which base stations are suspected to be an additional cause [17, 93–96].

A similar argument can be drawn from the discrepancy between the diffusion of alarmist media reports, the prevalence of EMF-related worries in the general population, and the actual number of EHS persons. In the Netherlands in 2010, for instance, 24% of persons aged 15 and over declared themselves very or fairly concerned by the potential health risks of EMF [97], but only one EHS support group was active with around 300 members [7]. In France, the only EHS support group existing at this time was about the same size, while the levels of concern were even higher, reaching 49% on a similar sample [97]. One may then argue that the cognitive hypothesis rests on a confusion between opinions, easily expressed in research settings but remaining inconsequential in daily life, with beliefs, held with sufficient conviction to alter subjective experience and behaviors. Transitioning from the former to the latter because of media reports alone would require exceptional levels of suggestibility, as acknowledged by recent studies exploring the role of personality traits such as absorption [98], that EHS persons have not been proved to have.

Conversely, the role of the mass media should not be underestimated. While they generally fail to change personal beliefs, they can influence opinions through “agenda-setting” and “framing” effects [86]: they do not affect what people think, but what people think *about*. Therefore, it is not surprising that they contributed decisively to the controversy surrounding EMF health effects [99] or that media coverage is associated with the prevalence of EHS [21]. People obviously need to know about EMF to attribute symptoms to EMF exposure, as they need to know about EHS to regard themselves as such. That this knowledge makes their attributions conceivable does not mean it causes their symptoms.

### Third limitation: EHS persons’ trajectories and experience

The last limitation of the cognitive hypothesis is its inconsistency with EHS persons’ trajectories and experience, as documented by qualitative studies [7–9]. In most cases, these trajectories do not begin with the reception of alarmist information on EMF health effects, but with the onset of an illness for which EHS eventually appears as an explanation. Most of the time, EHS persons even ignored the existence of EMF until after they got sick and learned about EHS. In the remaining cases, EHS persons had been warned against EMF harmfulness, but did not start to feel personally concerned until they fell ill: their worries remained abstract, as those of activists against the siting of mobile phone base stations, but eventually became a resource to make sense of unforeseen health problems.

Additionally, knowing whether one is currently exposed to EMF proves quite difficult, as the human body is devoid of sensory receptors to EMF. EHS persons must resort to vicarious techniques involving visual observation (e.g., of base stations on rooftops or cellphones in the hands of bystanders), informants (e.g., neighbors about EMF-emitting devices they use in their homes or building biologists), or dedicated EMF meters [100]. Their systematic implementation requires a careful monitoring of the environment that is impossible to maintain for long. As a result, EHS persons tend to assess their exposure only when they experience symptoms and feel the need of an explanation. Their attributions are essentially retrospective in nature and cannot cause symptoms they usually follow. Thus, EHS should not be confused with other environmental intolerances, especially multiple chemical sensitivity, which are triggered by stimuli that can be perceived sensorially, without the need of vicarious techniques requiring an active attention, and are more likely to involve unconscious conditioning mechanisms [9].

## The attributive hypothesis

Considering both its strength and limitations, the cognitive hypothesis may appear as incomplete at best, or misguided at worst. It can explain how EHS symptoms develop once negative expectations towards EMF have been acquired; however, the acquisition of such expectations remains unaccounted for, and with it, the initial step of the self-reinforcing process supposedly leading to EHS. A third hypothesis was thus proposed to explain the onset of EHS symptoms, rather than their persistence of aggravation: it conceives EHS as a coping strategy for pre-existing conditions, whose attribution to EMF exposure makes them easier to cope with.

### First argument: EHS persons’ trajectories

This attributive hypothesis rests foremost on studies of EHS persons’ trajectories. In most cases, these do not attribute spontaneously their illness to EMF exposure [7–9]. They first seek, and fail to obtain, medical assistance: none of the doctors they consult offer them acceptable solutions, i.e., a convincing diagnosis, preferably allowing for an effective treatment. This situation has distressing effects. The lack of a diagnosis casts doubt on the reality of their somatic perceptions and the legitimacy of their illness behaviors, making them feel profoundly stigmatized, as it raises the suspicion of a mental disorder [101]. The lack of an effective treatment exacerbates the feelings of anxiety and helplessness towards a disabling condition, especially when its nature remains unknown. This situation typically results from the medically unexplainable nature of EHS persons’ symptoms, for which no organic cause can be found despite multiple clinical investigations. It increases their receptivity to unconventional medical solutions and explains why (1) they consider the possibility they may have EHS once they hear about it, (2) they go through the learning process necessary to operationalize this attribution (e.g., to gain the ability to identify and avoid sources of EMF exposure), (3) they keep adhering to it once they discover its negative consequences (e.g., the high financial and social cost of EMF avoidance behaviors). Their *commitment* towards EHS rests on this attribution being the only solution they discovered to understand and manage their illness.

In the remaining cases, EHS persons receive prior information about EMF harmfulness, without feeling personally concerned. When abnormal symptoms onset at a later date, they instantly suspect EMF. However, they are only convinced of their responsibility once they notice that reducing their EMF exposure alleviates their symptoms, i.e., that EHS as a category allows them to conceive an effective coping strategy. Contrary to other EHS persons, they do not seek medical assistance for their symptoms, which are not necessarily medially unexplained (even though they do not appear, on average, less severe or disabling) [9].

These observations indicate that EHS persons are *motivated* to consider themselves as such, because it provides them with significant coping resources (e.g., a blame-free explanation, a way to predict and manage symptoms, social support from EHS groups, a victim identity, etc.). They echo an argument EHS persons frequently make: that they were convinced on practical grounds, when they noticed, to put is simply, that EHS works. This motivational dimension is notably absent from the cognitive hypothesis, especially from the comprehensive model of EHS presented above, which reduces cognition to a purely representational activity while neglecting its orientation towards the satisfaction of practical aims [102]. Yet, it explains why people who are not sick or deprived of medical solutions have no reason to feel personally concerned by the discovery of EHS, and more broadly, by alarmist media reports on alleged EMF health risks.

### Second argument: similarity between EHS and other functional somatic syndromes

The attributive hypothesis does not directly explain the origins of EHS symptoms but makes this problem somewhat less pressing. Indeed, many symptoms remain medically unexplained: depending on the estimates, they account for 10 to 33% of presenting complaints of new outpatients in primary care and 35 to 53% in secondary care, so that the category “signs, symptoms and ill-defined conditions” of the International Classification of Diseases is the fourth frequently used by general practitioners in the UK and the fifth most frequent reason for visiting a doctor in the USA [103]. These symptoms are predominantly transitory: less than half last more than a year, and even fewer lead to repeated consultations. Those that persist, however, tend to have severe consequences. They also remain predominantly unexplained, even when they occur concurrently with well-known somatic diseases. As a consequence, there is a high number of patients looking for diagnoses for their medically unexplained symptoms: the prevalence of somatoform disorders, defined by the persistence of such symptoms over 6 months, associated with a significant impairment or distress, was estimated to reach 6% in the general population, 16% in primary care patients and 33% in secondary care patients [103].

That situation offers an alternative explanation for the association between non-specific physical symptoms and perceived EMF exposure observed in environmental studies [58]: rather than feeling sick because they believe they are exposed to harmful EMF, their subjects might believe they are exposed to harmful EMF because they feel sick, and are looking for possible causes. This interpretation is coherent with the results of a recent prospective study, where increases in non-specific symptoms were not preceded by increases in perceived EMF exposure [66].

Additionally, people suffering from such symptoms may get diagnosed or diagnose themselves with various “functional somatic syndromes”, i.e., controversial and unexplained illnesses defined either by a predominant complaint (diffuse pain for fibromyalgia, asthenia for chronic fatigue syndrome, digestive problems for irritable bowel syndrome, etc.) or by an unproven etiology (participation in the Gulf War for the eponymous syndrome, exposure to chemical products for multiple chemical sensitivity, persistent Borrelia infection for chronic Lyme disease, etc.) [104]. The attributive hypothesis contends that EHS should be regarded as yet another functional somatic syndrome, based on especially contentious attributions. This view is supported by further similarities between EHS and these syndromes, namely, that they primarily affect women (who represent 62 to 95% of EHS subjects in questionnaire studies [1–6]) and are significantly comorbid with anxiety and depression [103, 105]. Consequently, EHS symptoms do not have to be explained on their own, as they could result from the variety of physio- and psycho-pathological mechanisms supposedly involved in functional somatic syndromes (e.g., autonomic imbalance for asthenia, central sensitization for pain, etc.) [106].

### First limitation: weaknesses of qualitative methods

The attributive hypothesis rests mostly on qualitative interview data, which suffer from significant weaknesses. These first relate to possible bias, affecting notably memory or desirability. The former results from the tendency to remember more easily and perceive as more important past events that are congruent with present beliefs. The latter depends on the propensity to favor answers making a positive impression on the interviewer, e.g. for EHS persons, by failing to mention their psychological distress. Selection bias are also likely to occur, as only specific individuals may consent to an interview. Furthermore, qualitative interview data can only shed light indirectly on unconscious aspects of cognitive activity: they are more appropriate to study the reasons put forward by the subjects rather than the true causes of their beliefs and behaviors, which are not necessarily identical, especially in apparent cases of irrationality [107]. They are also difficult to analyze rigorously because of their heterogeneity, reflecting the richness and diversity of illness experience in modern societies [108]. As a consequence, their interpretation always remains somewhat arbitrary, so that the reconstructed individual trajectories might become precarious. Lastly, qualitative interview data are expensive to produce and can only be collected from small samples, whose representativity cannot be guaranteed: the conclusions drawn from them are vulnerable to the contingencies of recruitment and difficult to generalize. Qualitative studies of EHS persons’ trajectories would thus benefit from being replicated by different researchers in different contexts, while using innovative interview and analytical methods to enhance the reliability of their results.

### Second limitation: explaining the choice between competing attributions

Given the high prevalence of medically unexplained symptoms, and the variety of attributions available to make sense of them, the attributive hypothesis faces another challenge: how to explain that so few of the potentially concerned patients end up diagnosing themselves EHS? Why they, and what happens of the others? Do they choose another attribution, receive the diagnosis of a functional somatic syndrome, or remain deprived of any explanation for their symptoms? For what reason? These questions remain essentially unaddressed, as no comparative study has been conducted of the experience and trajectories of patients endorsing different attributions for their medically unexplained symptoms. A pilot study took place in France in 2016–17, involving a comparison between persons with EHS or fibromyalgia, whose global results await full publication [110].

## Conclusion

None of the reviewed hypotheses proves totally acceptable. The debate on the origins of symptoms experienced by self-declared EHS persons remains open, even though its terms and implications are hopefully clearer. Considering available evidence, three avenues for future research can be envisioned that could help refine EHS explanatory hypotheses and decide between them, while filling significant voids in the scientific literature. The first would be to study systematically the symptoms, attributions, *and behaviors* of EHS persons to determine whether all three are connected, as supposed by the attributive hypothesis, or only the formers, as assumed by the cognitive hypothesis. What is at stake is whether opinions about EMF harmfulness and beliefs about EHS are distinct phenomena, that should be analyzed separately, and whether behaviors are a relevant indicator of commitment towards EHS. The second avenue would be to conduct clinical trials of cognitive and behavioral therapies aimed at symptom reattribution, which offers the best opportunity to alleviate EHS symptoms if the cognitive hypothesis is correct. Of note, four such trials were conducted in the late 1990s, whose results are too disparate to be interpreted as vindicating or refuting that hypothesis [109]. Today, given the aggravation of the controversy surrounding EHS, they would probably face severe acceptability challenges. The third avenue for future research, as exposed above, would consist in the rigorous comparison of experience and trajectories of people with EHS and other functional somatic syndromes. If the attributive hypothesis is valid, the only differences to appear should be circumstantial, i.e., EHS symptoms could as well have been attributed to other causes.

The first avenue may be the most urgent. Not knowing what is at the heart of the EHS phenomenon is a major source of uncertainty, which hinders its understanding as well as future research. Indeed, a possibility well worth considering is that all three explanatory hypotheses are true, because they apply to distinct forms of EHS. Such has been argued by several authors, who proposed to distinguish between (1) people declaring themselves sensitive to a few specific devices or to a wide array of exposure sources [75, 76], (2) people believing their health is negatively affected by EMF or also labelling themselves as EHS [59], or (3) people reporting a sensitivity to EMF or also registering with a militant group [5]. Available data indicates that the latter have more symptoms and a worse general health than the former, are more likely to seek treatment, are more worried about their health and report more environmental sensitivities. Therefore, their conditions might have different origins, with the cognitive hypothesis better accounting for the symptoms of mere “attributers” and the attributive hypothesis more suitable for “full-blown EHS” persons. Another distinction has been proposed between three categories of people: “cautious but not sick” (e.g., New Agers with modern health worries or protesters against the siting of base stations), “sick but not cautious” (e.g., patients with non-specific physical symptoms that they tentatively attribute to EMF, especially in research settings, without trying to reduce their exposure), and “sick and cautious” (who are sufficiently convinced of EMF harmfulness, and sufficiently motivated by their ill health, to deliberately reduce their exposure at the price of altering their lifestyle) [9]. Whatever distinction is the most appropriate, if EHS is to be better understood, it seems inevitable to take a closer look at what it is actually made of.

## Data Availability

Not applicable.
